# Effects of bioelectricity generation processes on methane emission and bacterial community in wetland and carbon fate analysis

**DOI:** 10.1186/s40643-022-00558-8

**Published:** 2022-06-20

**Authors:** Shentan Liu, Hongpu Xue, Yue Wang, Zuo Wang, Xiaojuan Feng, Sang-Hyun Pyo

**Affiliations:** 1 College of Geology and Environment, Xi’an University of Science and Technology, Xi’an, 710054 Shaanxi China; 2 Biotechnology, Department of Chemistry, Faculty of Engineering, Lund University, 22100 Lund, Sweden; 3 School of Environment, Tsinghua University, Beijing, 100084 China; 4 School of Water and Environment, Chang’an University, Xi’an, 710054 Shaanxi China

**Keywords:** Constructed wetland, Microbial fuel cell, Greenhouse gas, Methane, Fate pathway

## Abstract

Wetlands are an important carbon sink for greenhouse gases (GHGs), and embedding microbial fuel cell (MFC) into constructed wetland (CW) has become a new technology to control methane (CH_4_) emission. Rhizosphere anode CW–MFC was constructed by selecting rhizome-type wetland plants with strong hypoxia tolerance, which could provide photosynthetic organics as alternative fuel. Compared with non-planted system, CH_4_ emission flux and power output from the planted CW–MFC increased by approximately 0.48 ± 0.02 mg/(m^2^·h) and 1.07 W/m^3^, respectively. The CH_4_ emission flux of the CW–MFC operated under open-circuit condition was approximately 0.46 ± 0.02 mg/(m^2^·h) higher than that under closed-circuit condition. The results indicated that plants contributed to the CH_4_ emission from the CW–MFC, especially under open-circuit mode conditions. The CH_4_ emission from the CW–MFC was proportional to external resistance, and it increased by 0.67 ± 0.01 mg/(m^2^·h) when the external resistance was adjusted from 100 to 1000 Ω. High throughput sequencing further showed that there was a competitive relationship between electrogenic bacteria and methanogens. The flora abundance of electrogenic bacteria was high, while methanogens mainly consisted of *Methanothrix*, *Methanobacterium* and *Methanolinea*. The form and content of element C were analysed from solid phase, liquid phase and gas phase. It was found that a large amount of carbon source (TC = 254.70 mg/L) was consumed mostly through microbial migration and conversion, and carbon storage and GHGs emission accounted for 60.38% and 35.80%, respectively. In conclusion, carbon transformation in the CW–MFC can be properly regulated via competition of microorganisms driven by environmental factors, which provides a new direction and idea for the control of CH_4_ emission from wetlands.

**Graphical Abstract:**

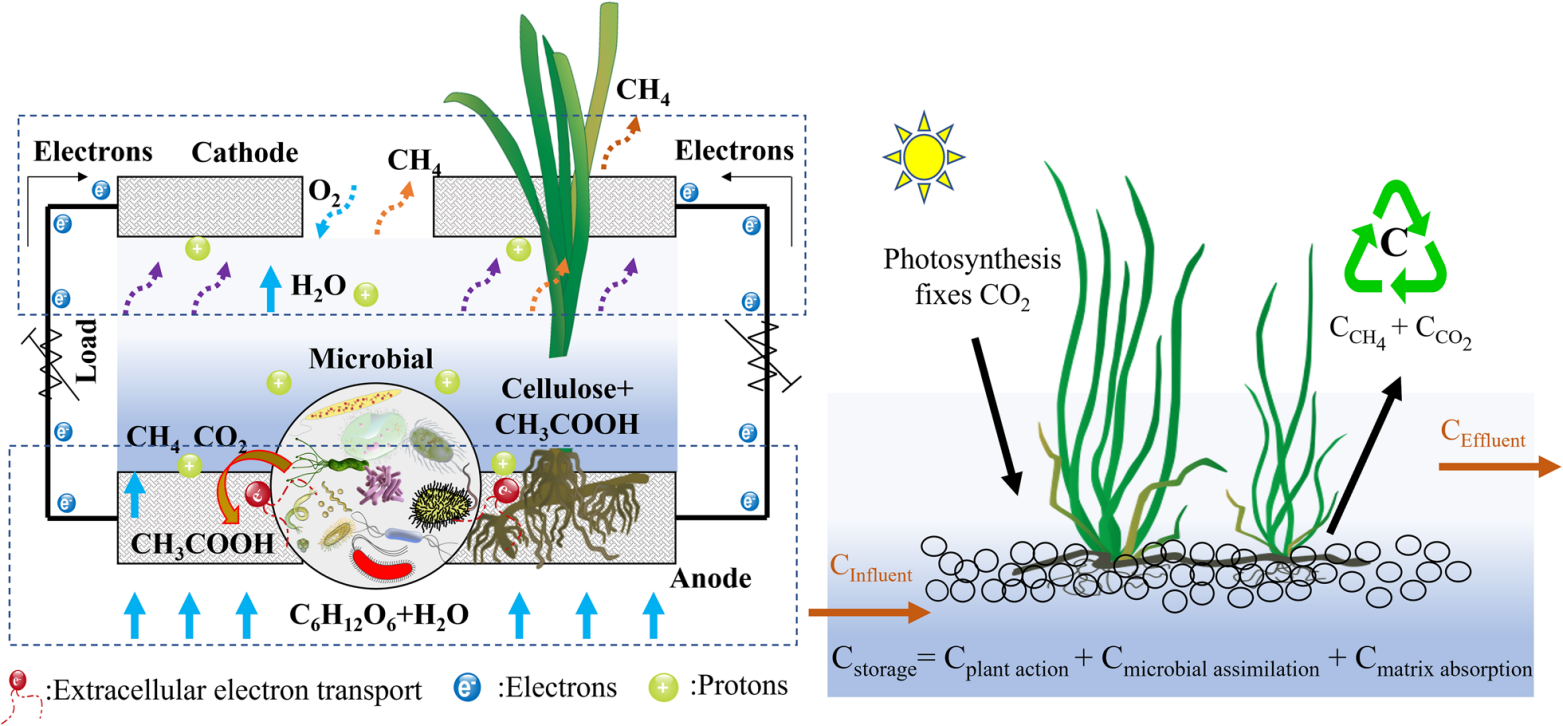

## Highlights

1. Effects of different operating conditions on CH_4_ emission.
2. The competitive relationship between electricigens and methanogens was analysed.
3. The morphology and content of C element in different phases were discussed.
4. The bacterial population structure under different conditions was analysed.
5. The mechanism of CH_4_ emission from CW–MFC was described in detail.

## Introduction

In recent years, the generation and release of greenhouse gases (GHGs, such as CO_2_, CH_4_ and N_2_O) have led to a sharp rise in global temperature. The annual growth rate of CO_2_ is approximately 2 ppm per year, and the concentration of CO_2_ is expected to rise to 800–1000 ppm by the end of the twenty-first century (Lopez-Pacheco et al. 2021; Yu and Chen 2019). In the carbon cycle, the warming effect of CH_4_ is 28–36 times greater than that of CO_2_, which makes it urgent to control CH_4_ emissions (Riddick et al. 2019). Atmospheric CH_4_ mainly comes from wetlands (including natural wetlands and constructed rice field wetlands), which account for approximately 20–40% of global CH_4_ emissions (Bloom et al. 2010; Oshita et al. 2014). Therefore, it is significant to study the characteristics and influence mechanisms of CH_4_ emissions from wetland ecosystems, and then formulate corresponding GHGs (mainly CH_4_) emission reduction measures on this basis to alleviate the global warming effect.

Constructed wetland (CW) is an extremely mature wastewater treatment technology that removes pollutants from wastewater through microbial metabolism, plant physiological activity, matrix adsorption, sedimentation and filtration (Wu et al. 2017). Due to low cost, strong decontamination and less secondary pollution, CWs have become a popular wastewater treatment method and are used on a large scale (Mohammed et al. 2021). However, the use of CWs for sewage treatment may increase CH_4_ discharge and lead to “pollution exchange”, so researchers have taken great interest in managing CWs and flooded rice fields to minimize CH_4_ emissions (Pangala et al. 2010). At this stage, a large number of studies have focused on various factors (e.g., irrigation, seasonality, fertilization and crop rotation) affecting CH_4_ emission in paddy wetlands and found that the average CH_4_ emission fluxes reached approximately 25–300 Tg/year (Wang et al. 2018; Xu et al. 2020). GHGs from wetlands are mainly affected by anaerobic microorganisms in the bottom layer of wetlands, and methanogenic bacteria have easy access to root cellulose for their own needs, resulting in large amounts of CH_4_ production (Liu et al. 2017; Zhang et al. 2021b). So far, the mechanism of CH_4_ emission and the impact of microorganisms on CH_4_ production have rarely been reported. Moreover, it is urgently necessary to explore new strategies to control CH_4_ emissions in the wastewater treatment process by CWs.

Microbial fuel cells (MFCs) are devices that use “microorganisms” as catalysts to degrade organic pollutants in wastewater and convert chemical energy into electrical energy (Catal et al. 2019). The degradation of organic pollutants in wastewater by MFCs has become a reality and is gradually maturing. In bio-electrochemical methods, CH_4_ can be collected from the biocatalyst of microbial electrolysis cells (MECs) not only by CO_2_ electro-conversion methanogenesis, but also by enriching microorganisms on the anode in anaerobic digestion via microbial electrosynthesis (applied voltage) to increase CH_4_ production (Flores-Rodriguez and Min 2020; Zhang et al. 2019). It has been reported that CH_4_ production reduces coulomb efficiency (CE) and thus the sensing accuracy of MFCs, but the significance of such suppression can only be specific to application (Kaur et al. 2014). Wetlands are the main source of long-lived GHGs, and it is possible to couple CWs with MFCs to control CH_4_ emissions, which provides a technological advantage in suppressing CH_4_ emissions. Most of previous studies have been conducted by controlling the operating conditions of the reactor or adding inhibitors (e.g., antibiotics) to observe the CH_4_ emission flux (Xu et al. 2021b). However, the real reasons for controlling CH_4_ emission should be synthetically analyzed from the mechanism of gas emission and bacterial community, which need to be further explored. As previously reported, microorganisms in an electroactive constructed wetland system are often restricted by carbon source and carbon/nitrogen ratio (COD/TN) (Xu et al. 2021a). In addition, plant roots play vital roles in CH_4_ emission, and it can not only provide microbial nutrients (e.g., plant photosynthate and cellulose) for the growth of microorganisms without the addition of carbon source (Liu et al. 2013), but also discharge gases into the environment through its vascular tissue. So far, the bio-electrochemical mechanisms of controlling CH_4_ emission are not fully understood, which need further in-depth discussion from the perspective of mechanism and bacterial community analysis. Furthermore, it is crucial to explore microbial competition strategies driven by environmental factors to regulate CH_4_ emissions.

In this experiment, emergent plants with strong hypoxia tolerance were planted in the anode compartment, and the static box method was used to cover the wetland for collecting greenhouse gases. The objectives were: (1) to explore different operating conditions and configurations on GHG emissions, (2) to analysis the interrelationship between gas emissions and electricity production, (3) to summarize the minimum control conditions for CH_4_ emissions, (4) to explore the final fate of the carbon, (5) to further analysis of bacterial community structure under different conditions, and (6) to further analyse the mechanism of CH_4_ emission.

## Methods

### Reactor construction

**Fig. 1:**
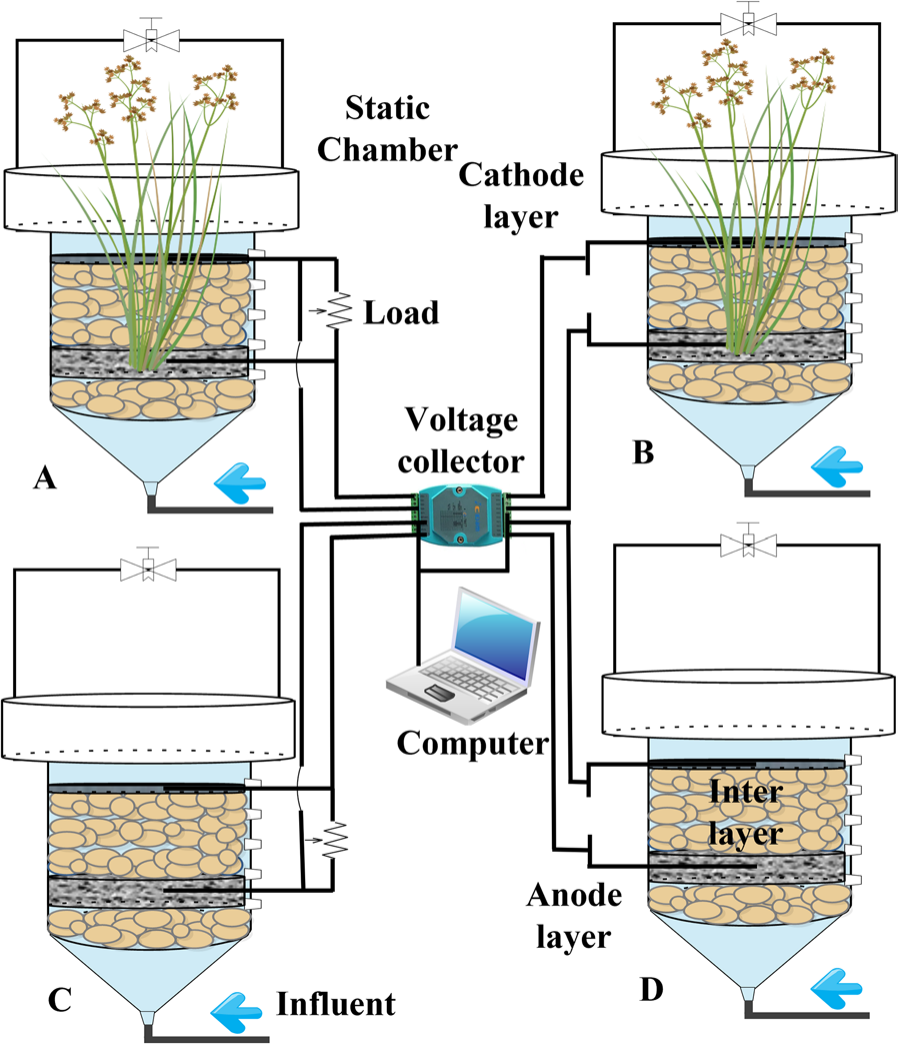
Configuration of the CW–MFC. Closed-circuit/with plants group (group **A** CCP), open-circuit/with plants group (group **B** OCP), closed-circuit/without plants group (group **C** CCN), and open-circuit/without plants group (group **D** OCN)

A CW–MFC made of acid- and alkali-resistant polypropylene plastic columns was constructed. Four groups of reactors were designed for this experiment, as shown in Fig. 1.

The total height of the system body is 100 cm. The main reactor is 55 cm in height, while the height of the reaction chamber is 45 cm and the height of the static box is set at 45 cm. The total volume of the reactor is 6.9 L, and the effective water storage volume is approximately 1.0 L. The lower part of the reactor is equipped with a conical inlet chamber with a water distributor, and the lower part of the chamber is filled with 5 cm thick gravel (particle size: 1–3 cm) as a support layer. The bioanode material is carbon fiber felt (CFF, thickness: approximately 2.5 cm) with stainless steel wire mesh (SS, wire diameter: 2 mm, pore size: 0.5 mm) sandwiched between the CFF to collect electrons. The intermediate layer was filled with gravel to a height of 10 cm. The electrode material of the air cathode layer is the same as that of the anode, and the anode and cathode are connected by titanium wire leading out of the reactor and connected by alligator clip wires, with a 1000 Ω resistance wire connected at both ends as the starting condition. After the CW–MFC start-up was completed and stable, the open and closed-circuit reactors were tested. The upper side is equipped with an overflow tank, the purpose of which is to put in the static box and pour in deionized water to achieve the condition of water seal and avoid gas exchange with the outside atmosphere. In this experiment, a new type of inter-root-anode CW–MFC was constructed by selecting a water-holding wetland plant *Acorus calamus* with high decontamination ability, high anoxic tolerance and cold tolerance planted in the bioanode.

### Inoculation and system operation

Anaerobic sludge was taken from a wastewater treatment plant and incubated anaerobically for 2–3 days. The cultured anaerobic sludge was inoculated into the anode, and then nutrient solution was injected from the lower inlet by a peristaltic pump. The nutrient solution was configured as follows: 5 mM phosphate buffer solution (PBS), 0.2 g/L C_6_H_12_O_6_, 0.15 g/L NH_4_Cl, 0.13 g/L KCl, 3.13 g/L NaHCO_3_, and 1 ml/L micronutrient solution (Liu et al. 2014). After inoculation, both electrode ends of the reactor were led by titanium wires to the voltage acquisition board, and cell voltage data were recorded by the computer (once in 600 s). The start-up time was approximately 30 days, and the nutrient solution was replaced if the voltage dropped to 50 mV. The maximum voltage and stable operation mean the completion of CW–MFC start-up.

### Water quality and bioelectricity generation performance determination

All samples were filtered through 0.45 μm filter membrane to remove suspended solids. COD was measured using the potassium dichromate method (APHA method 5220). The COD removal efficiency (RE_COD_) was calculated according to the following equation:

1 $${\mathrm{RE}}_{\mathrm{COD}}=\frac{{\mathrm{COD}}_{\mathrm{in}}-{\mathrm{COD}}_{\mathrm{eff}}}{{\mathrm{COD}}_{\mathrm{in}}}\times 100\%$$

where COD_in_ and COD_eff_ represent influent COD concentration and effluent COD concentration (mg/L), respectively.

Total organic carbon (TOC) was tested by combustion oxidation–non-dispersive infrared absorption method (HJ 501-2009), which responded to the total amount of carbon contained in dissolved organic matter in water bodies. There are differential subtraction and direct methods for the measurement of total organic carbon, and the differential subtraction method was chosen for the calculation of TOC in this study, as shown in the following equation:

2 $$\mathrm{TOC}=\mathrm{TC}-\mathrm{TIC}$$

where TC and TIC represent total carbon and total inorganic carbon, respectively.

Once the electricity performances of the CW–MFCs were stable, cell voltage data were mainly recorded by the acquisition board (DAQ3323, 6-digit sensitivity) and delivered to the computer. Power density curves and polarization curves were measured by regulating external resistance method, i.e., using a resistance box (XJHS1000) to change the resistance from 9000 to 100 Ω, and the time step was 5 min to ensure the stabilization of CW–MFC voltage under low dynamic conditions (Degrenne et al. 2012).

The coulomb efficiency (CE) reflects the ratio of the actual cell yield to the theoretical cell yield, and it can evaluate the superiority of the MFC power production performance. The specific formula is shown in the following equation:

3 $${\text{CE}} = \frac{I}{{F\left( {4 \div 32} \right)\Delta {\text{COD}} \times Q_{{{\text{In}}}} }}$$

where CE is coulombic efficiency of the CW–MFC (%), *I* is current (A), *F* is the Faraday constant (96,485 C/mol), *Q*_In_ is flow rate (L/s), *∆*COD is the difference of COD between influent and effluent (g/L), 4 is the number of electrons obtained by oxidation–reduction of 1 mol oxygen, 32 is the molar mass of oxygen, 32 g/mol.

### CH_4_ collection, determination and flux calculation

Gas was collected by the static box method, with gas collection holes on top of each box (Liu et al. 2017). Samples were taken by introducing one end of the suction pump into the static chamber and the other end into the gas bag. CH_4_ and CO_2_ were measured using a gas chromatograph (Agilent 6890B, USA) equipped with a FID detector and a TCD detector, and the carrier gas was nitrogen. The gas samples were collected at 8–10 am each day. For the accuracy of the experimental data, the gas was sampled in triplicate each time. The actual gas production was calculated according to the following equation:

4 $$J=\frac{\mathrm{d}c}{\mathrm{d}t}\frac{V}{A}\frac{\mathrm{MP}}{\mathrm{RT}}$$

where *J* is the emission flux of CH_4_ (g/m^2^·h), *V* is the effective volume of closed chamber for collecting gas (0.012 m^3^), *A* is the opening surface area of the reactor (0.015 m^2^), d*c*/d*t* is the concentration change of CH_4_ in the closed chamber per unit time (mL CH_4_/mL gas/min), *T* is average temperature (25 ℃, 298.15 K), *M* is the molar mass of CH_4_ (16 g/mol), *P* is the actual atmospheric pressure (101,325 Pa), *R* is the gas equilibrium constant (8.314 J/mol/k).

### Measurement and calculation of element C

The proportions of carbon content in solid phase, liquid phase and gas phase were obtained by observing and analyzing the trend of carbon content before and after input in the system. The specific distribution is shown in Eqs. (5–7):

5 $${C}_{\mathrm{Influx}}={C}_{\mathrm{Outflux}}$$

where *C*_influx_—total external input carbon content; *C*_Outflux_—total carbon content in different fate pathways:

6 $${C}_{\mathrm{Influx}}={C}_{\mathrm{Influent}}+{C}_{\mathrm{Photosynthesis}}$$

where *C*_Influent_—carbon content of liquid phase entering the system; *C*_Photosynthesis_—carbon content of plant photosynthesis entering the system:

7 $${C}_{\mathrm{Outflux}}={C}_{\mathrm{Liquid}}+{C}_{\mathrm{Gas}}+{C}_{\mathrm{Solid}}+{C}_{\mathrm{Others}}$$

where *C*_Liquid_—carbon content of liquid phase in effluent; *C*_Gas_—flux of CH_4_ and CO_2_ produced in the system; *C*_Solid_—carbon content transferred or converted through matrix absorption, microbial assimilation and plant physiological activities in the system; *C*_Others_—uncounted carbon contents.

### Bacterial and archaeal communities analysis

Illumina sequencing analysis of microbial communities was performed by sampling anodic CFF from open-circuit planted CW–MFC, closed-circuit planted CW–MFC and closed-circuit non-planted CW–MFC (Sequencing service was provided by Shanghai Personal Biotechnology Co., Ltd., China). After extracting microbial genomic DNA from anodic CFF, the upstream primer 338F (primer sequence: ACTCCTACGGGAGGCAGCA) and the downstream primer 806R (GGACTACHVGGGTWTCTAAT) were analysed to amplify the standard 16S V3–V4(a) region of bacteria. Then the PCR amplification of methanogenic bacteria was continued, and the mcrA gene of methanogenic bacteria was amplified by the upstream primer mcrA-F (GGTGGTGTMGGATTCACACARTAYGCWACAGC) and the downstream primer mcrA-R (TTCATTGCRTAGTTWGGRTAGTT). Microbial sequencing mainly used macrogenomic DNA extraction–OMEGA and PCR to amplify bacterial 16S rDNA functional gene fragments. The bacterial data were analyzed using the online platform Personalbio GenesCloud (https://www.genescloud.cn).

## Results and discussion

### CH_4_ emission fluxes from open/closed circuit CW–MFCs

**Fig. 2:**
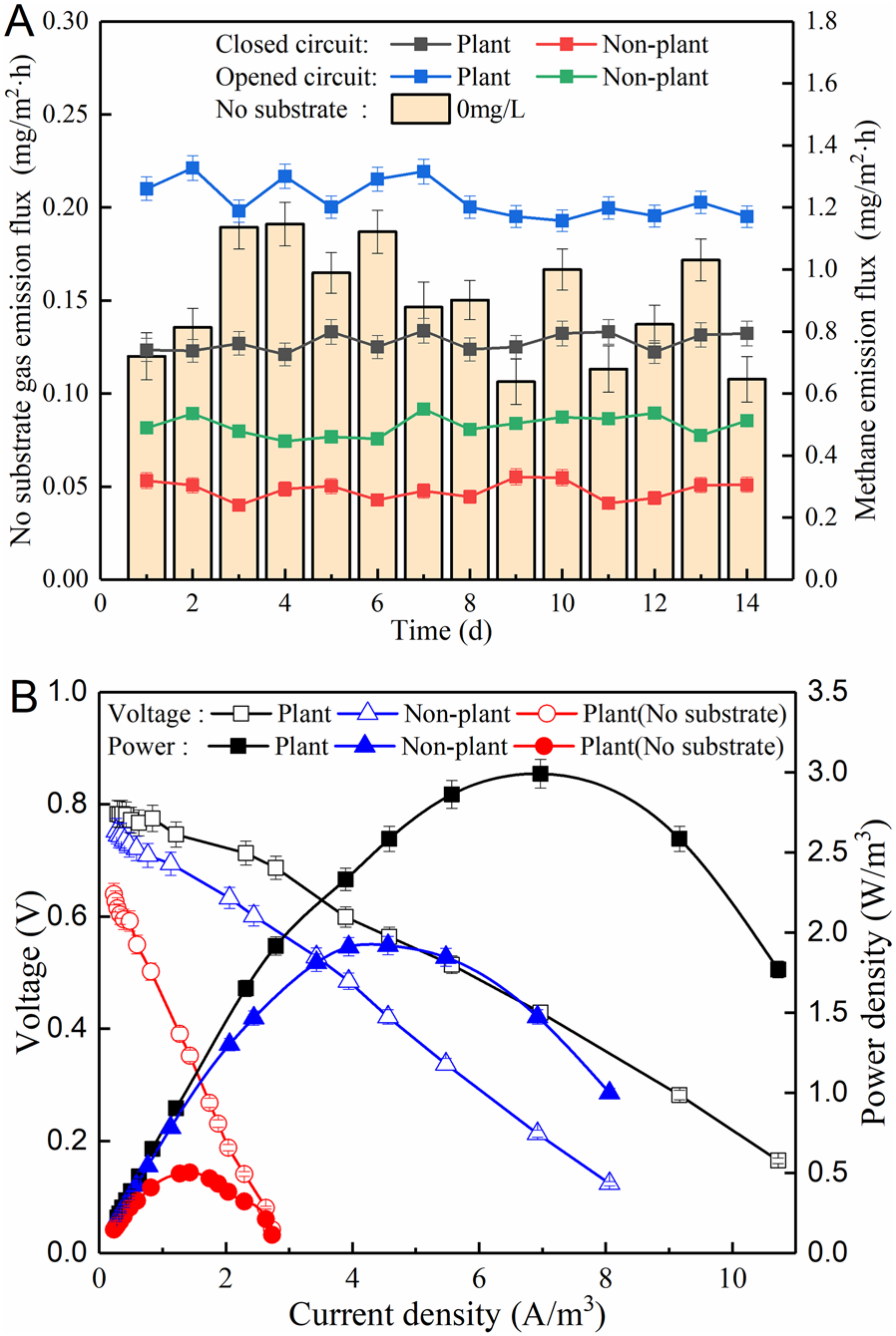
(**A**) Changes of CH_4_ emission flux and (**B**) polarization/power density curves of closed/open circuit CW–MFCs

The open/closed circuit model is one of the most important factors affecting CH_4_ emissions. In the open-circuit case, the CW–MFC is equivalent to a CW, so it is important to explore the difference between the open and closed-circuit modes of CW–MFCs, the dynamics of CH_4_ emission during operation are shown in Fig. 2A. The CH_4_ emission flux from the open-circuit planted CW–MFC was 0.46 ± 0.02 mg/(m^2^·h) higher than that from the closed-circuit planted CW–MFC, while in the non-plant systems, the CH_4_ emission flux under the open circuit mode was higher than that under closed circuit mode by approximately 0.21 ± 0.01 mg/(m^2^·h), indicating that microbial electrogenesis had inhibitory effect on CH_4_ emission. In previous study, CH_4_ emission fluxes were 6.37–7.28 and 7.43–8.36 mg/(m^2^·h) for closed circuit and open circuit CW–MFCs, respectively, and the CH_4_ emission fluxes in our study was basically consistent with the relevant literature report (Xu et al. 2021b). The CH_4_ emission difference between the open/closed circuit models is mainly caused by the bioanode. It has been reported in the literature that electrons are more easily produced in closed circuit mode due to electrical stimulation enhancing the growth of electrochemically active bacteria (EAB), and with the same source of carbon and nitrogen, the EAB have easier access to food, allowing methanogenic bacteria to be suppressed (Kaur et al. 2014). In an anaerobic fermentation environment (i.e., the open circuit CW–MFC), as no current passes, methanogenic bacteria proliferate and thus increase the production of CH_4_ gas (Ishii et al. 2008).

**Fig. 3:**
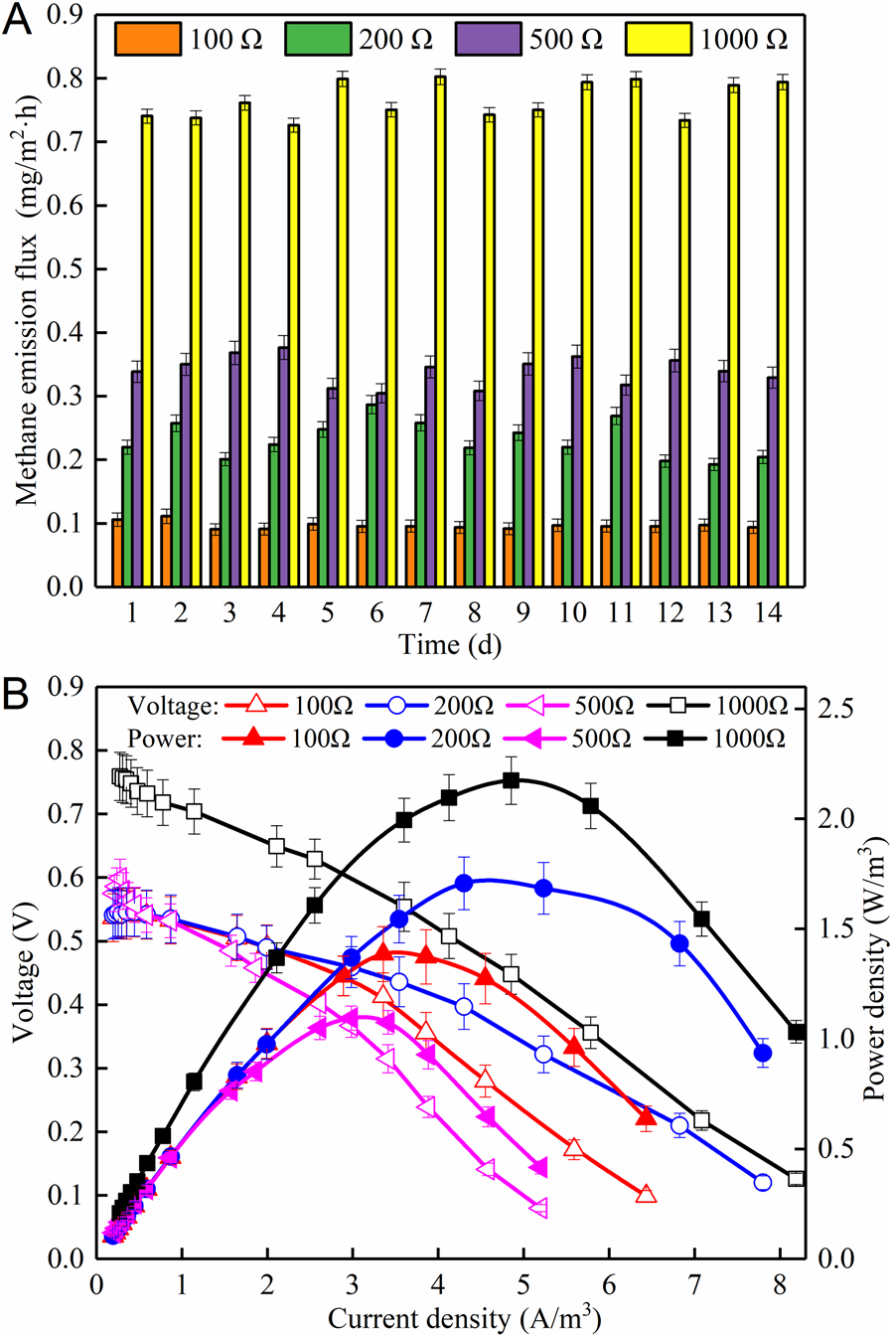
(**A**) CH_4_ emission fluxes and (**B**) polarization/power density curves of CW–MFCs with different external resistors

The emission flux of CH_4_ was extremely influenced by wetland plants as well as substrate concentration in the solution (Rahmani et al. 2022). Under influent glucose concentration of 200 mg/L and closed-circuit operation mode, the CH_4_ emission flux from the plant group increased by approximately 0.48 ± 0.02 mg/(m^2^·h) compared to the non-plant group. Plant root exudates (or rhizosphere deposits) can be easily decomposed by microorganisms for their own consumption, thus increasing the CH_4_ emission (Rismani-Yazdi et al. 2013; Sun et al. 2021). Moreover, in the presence of wetland plants, GHG emissions were not only in the form of bubble ebullition and liquid-phase diffusion, but also in the form of gas transport through plant aerenchyma (Waldo et al. 2019). Excluding the effect of substrate (i.e., substrate concentration of 0 mg/L) as shown specifically in Fig. 3A, the average CH_4_ emission flux of 0.15 ± 0.01 mg/(m^2^·h) was found for the plant group under closed circuit condition. The fact that cellulose (e.g., exfoliated root tissue) can be used as a carbon source by *Cellulomonas fimi*, *Cellulomonas biazotea* and C*ellulomonas flavigena*, and *Cellulomonas* spp. is a direct cellulose-based microorganism (Takeuchi et al. 2017; Toczylowska-Maminska et al. 2018). However, in the absence of substrate, the microorganisms on the anode of the plant group had no sufficient nutrients, and their quantity and activities would decrease to a certain extent, thus leading to less CH_4_ production and emission.

The relationship between CH_4_ emission and output voltage can be further illustrated with polarization curves and power density curves. As shown in Fig. 2B, the power density of the plant group was 1.07 W/m^3^ higher than the non-plant group, corresponding to an internal resistance of 187.02 and 257.91 Ω, respectively. Previous studies indicate that plant roots can provide more terminal reduction electron acceptors (i.e., O_2_) and increase the reduction medium on the cathode surface, thus reducing the internal resistance of the system and increasing the output voltage (Liu et al. 2022; Nandy et al. 2019). However, the plant caused an increase in the CH_4_ emission flux. From a microbiological point of view, EAB (e.g., *Aspergillus*, *Actinobacter*, *Fimicus* and *Acidobacter*) on the anode compete with methanogenic bacteria for nutrients and become the dominant flora, thus resulting in increased voltage and decreased CH_4_ production. In general, the transport of CH_4_ gas through plant vascular increases the CH_4_ emission. According to the literature, rice (*Oryza sativa*), a typical vascular plant in paddies, induced the emission of CH_4_ as well as CO_2_ (Gupta et al. 2021). The current density and power density of the plant group without substrate reached only 1.43 A/m^3^ and 0.50 W/m^3^, respectively, yet the internal resistance was as high as 233.15 Ω. The reason for electricity decline might be a decrease in available organics as fuel, and the EAB do not have enough nutrients for their own needs, thus reducing the production of electrons and making it difficult for protons to pass from the anode to the cathode. It has been reported in the literature that cellulose can provide a certain amount of nutrient supply for the microorganisms in MFCs, especially the *Cellulomonas* strain NBRC-15513 (Khawdas et al. 2019).

### Influence of external resistance on CH_4_ emission from the CW–MFCs

Long-term application of external load impacts on anodic biofilm microarchitecture and biochemical metabolism (Pasternak et al. 2018), which in turn affect the performance of CW–MFC (e.g., voltage output variation and pollutant removal effect). To ensure the appropriate use of external resistance, different external resistances (e.g., low *R*_ext_ of 100 Ω, moderate *R*_ext_ of 200 Ω, high *R*_ext_ of 500 Ω and 1000 Ω, as the *R*_int_ of the CW–MFC was around 200 Ω) were set to observe bioelectricity generation and the control of CH_4_ emission in the CW–MFC. As shown in Fig. 3A, with the external resistance increasing, the emission trend of CH_4_ also increased gradually. Compared with external resistances of 100 Ω, the 1000 Ω resulted in increased CH_4_ emission by 0.67 ± 0.01 mg/(m^2^·h). This result indicates that the external resistance was in positive proportion to CH_4_ emission from the CW–MFC. It was further understood from Fig. 3A that different external resistances had different control over CH_4_ emission, mainly because microbial metabolic activities, substrate utilization kinetics and electron transfer rate were not identical under various external resistances conditions (Picioreanu et al. 2007). Previous study also demonstrates that the increase of external resistance results in enhanced growth of methanogens and inhibited growth of EAB, as the EAB mainly consume substrate to cultivate more microbes under the action of electrical stimulation (i.e., smooth electron transfer condition) (Picioreanu et al. 2008).

Table 1 presents estimations of CW–MFC performance operating at different external resistances. With the external resistance decreasing, the current density gradually increased and CH_4_ emission was well controlled. This result suggests that there is a strong competitive relationship between microbial electrogenesis and microbial methanogenesis (Liu et al. 2017). Therefore, it can be speculated that the lower external resistance facilitated the electron transfer from anode to cathode, thus providing growth superiority to the EAB (Pinto et al. 2011). Indeed, the increase of external resistance from 100 to 1000 Ω reduced the CE of the CW–MFC from 22.59 to 9.03%, and moreover, COD removal efficiency decreased from 95.71 to 90.12%. This might be because the high external resistance obstructed the electron transfer and reduced the ability of EAB to produce electrons, and the consumption of organic matter by anodic EAB was greatly reduced, thus increasing the emission flux of CH_4_. Notably, the CW–MFC with *R*_ext_ of 200 Ω obtained the lowest *R*_int_ of 166.16 Ω and the largest power density of 1.68 W/m^3^, which indicated that the appropriate applied external resistance close to *R*_int_ was beneficial to reducing the internal resistance and promoting the power output (Nikhil et al. 2018).

**Table 1:** Influence of external resistances on the long-term performance of the CW–MFCs

*R*_ext_ (Ω)	Voltage (V)	Current density (A/m^3^)	Power density (W/m^3^)	*R*_int_ (Ω)	CE (%)	RE_COD_ (%)	CH_4_ flux (mg/m^2^·h)
100	0.172	5.58	0.96	216.5	22.59	95.71	0.11
200	0.322	5.23	1.68	166.16	21.40	94.31	0.23
500	0.402	2.61	1.05	241.32	10.94	92.17	0.35
1000	0.649	2.11	1.37	248.55	9.03	90.12	0.78

Figure 3B shows that the polarization–power curves of CW–MFCs were also affected deeply by the long-term application of external resistances. The open-circuit voltage decreased gradually with the decrease of resistance (0.759, 0.575, 0.541 and 0.537 V for CW–MFCs with *R*_ext_ of 1000, 500, 200 and 100 Ω, respectively), which might be related to accelerated consumption and reduction of oxygen under high current conditions, as there was no gas exchange between the air-tight reactors and atmosphere. The highest maximum power density of 2.17 W/m^3^ was observed in CW–MFC operated with *R*_ext_ of 1000 Ω, followed by CW–MFC with *R*_ext_ of 200 Ω (1.70 W/m^3^), then CW–MFC with *R*_ext_ of 100 Ω (1.39 W/m^3^) and CW–MFC with *R*_ext_ of 500 Ω (1.09 W/m^3^).

External load is a vital factor affecting the generation and transportation of electrons in the CW–MFC. Too low external resistance leads to over-quick electron flow speed, far higher than the maximum sustainability, eventually resulting in substantial decline of power generation (Nikhil et al. 2018). However, excessive external resistance brings anode potential to more positive value and decreases the amount of EAB, which are deleterious to bioelectricity production and CH_4_ mitigation (Pinto et al. 2011). Therefore, in this study, the appropriate use of external resistance of 200 Ω resulted in the highest power density with less CH_4_ emission from the CW–MFC.

### The final fate of carbon in the CW–MFC

The question of the final fate of the wetland “carbon” is to explore the key processes of carbon conversion in wetlands, which have important implications for global carbon dynamics and carbon saving function. The experiments in this section described the migration and transformation of wetland carbon fractions between different interfaces (e.g., atmosphere, water body, matrix, roots and microorganisms) and summarized the carbon cycle in terms of the carbon stocks (i.e., carbon fate including the gas phase C, liquid phase C and solid phase C).

**Fig. 4:**
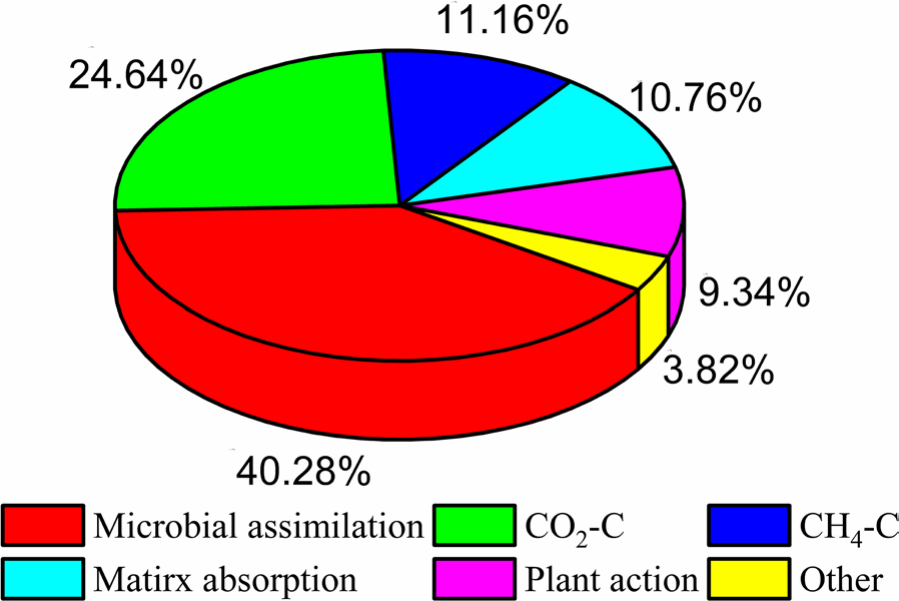
Ultimate fate of carbon in the carbon cycle of the CW–MFC

In a 14-day operation cycle, TC of 254.70 mg/L was removed from aqueous solution (TC_influent_ = 606.90 mg/L, TC_effluent_ = 352.20 mg/L) and mostly was transported and transformed through microbial metabolism under anaerobic conditions. Among them, the C content fixed by microbial assimilation was approximately 102.60 mg. In addition to microbial metabolism, wetland plants have an important influence on the transformation pathway of biogenic carbon in water, affecting the generation, emission and consumption of GHGs (i.e., CH_4_ and CO_2_) through various physiological and biochemical activities of plants (e.g., plant photosynthesis and rhizosphere effect). Of course, the aerenchyma tissues of plant roots can transmit a certain amount of GHGs to the atmosphere. The GHG emission C contents of CO_2_–C and CH_4_–C were approximately 62.76 mg and 28.42 mg, respectively. The C contents immobilized via matrix absorption and plant action (i.e., root absorption and plant photosynthesis) were approximately 27.40 mg and 23.80 mg, respectively, and the rest uncounted C contents accounted for approximately 9.72 mg (e.g., dewdrops on gas caps and reactor walls). As shown in Fig. 4, according to the statistical calculation of the carbon fate in the CW–MFC, the sequence of the carbon contents in different forms were as follows: microbial assimilation (40.28%) > CO_2_–C (24.64%) > CH_4_–C (11.16%) > matrix absorption (10.76%) > plant action (9.34%) > others (3.82%).

**Fig. 5:**
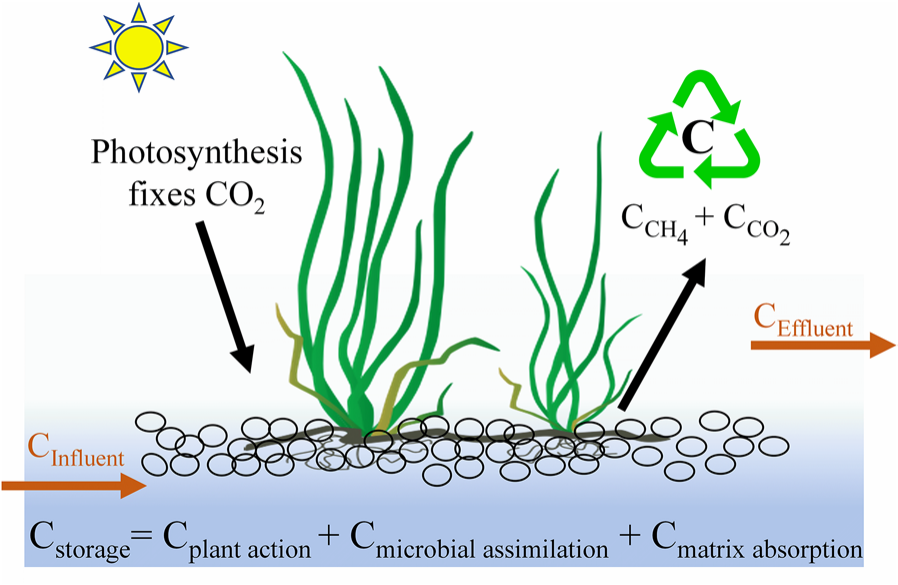
Carbon balance diagram in the CW–MFC

Figure 5 shows the carbon balance diagram, and the carbon balance model is a simplification of the carbon cycle process of CW–MFC micro-ecosystem, as shown in Eqs. (5–7). The exotic carbon of the CW–MFC was mainly organic carbon in the influent, which was transformed through different pathways including microbial assimilation, microbial metabolism, plant activity and matrix adsorption etc. Combined with the carbon fate results in Fig. 4, it can be concluded that the proportion of carbon fixation of the CW–MFC was approximately 60.38% (i.e., carbon storage, including plant action, microbial assimilation and matrix absorption), and the proportion of GHGs carbon emission was approximately 35.80% (i.e., CO_2_ and CH_4_ mainly produced by methanogens and EAB, respectively). Moreover, wetland plants converted gaseous CO_2_ into organic matter through photosynthesis. The above results suggest that wetland plays an important role as a carbon sink in the process of wastewater treatment, but it also inevitably releases a certain amount of GHGs.

In the CW–MFC, the production and emission of GHGs are mainly caused by microbial activities, and its pathways can roughly be divided into liquid–gas conversion and gas–gas exchange (Fig. 5). In addition to microorganisms, wetland plants also play crucial roles in CH_4_ emissions from the CW–MFC. The presence of wetland plants not only improves MFC electricity performance, but also leads to more CH_4_ emission mainly due to the transportation of CH_4_ via plant aerenchyma (Md Khudzari et al. 2019). Similarly, our research also found that the emission flux of CH_4_ from the non-plant CW–MFC tended to the lowest (Fig. 2A). Moreover, plant growth and activities may be affected by microbial electrogenesis, and a physiological plant study indicated that harvesting bioelectricity using plant–MFC promoted the photosynthetic rate of *Codiaeum variegatum* (Valdez-Hernández et al. 2022). Therefore, to more effectively control CH_4_ emission from the CW–MFC, it is necessary to further study the interaction mechanism between microbial electrogenesis and plant physiological activities in the future. According to the literature, due to the temporal and spatial variability of GHG emissions from wetlands, the emission flux of CH_4_ increases as the CO_2_ flux increases, mainly because the increase in CO_2_ concentration changes the concentration of oxygen in the root zone and the availability of carbon sources (Kao-Kniffin and Zhu 2013; Zhang et al. 2021a). Although wetlands are an important sink of exotic biogenic carbon (e.g., TOC in wastewater), biological activities (i.e., microbial and plant activities) and chemical phenomena are the key factors affecting the carbon dynamics in different phases. These conclusions should be highly regarded in the future research of carbon sinks in wetlands, as they might have important implications on wetland CH_4_ budgets as well as on global climate change.

### Bacterial community analysis

The data of bacterial community controlling CW–MFC GHGs emission are rather limited, so it is of great significance to analyze the richness and diversity of bacterial community (Lopez et al. 2019). As shown in Table 2, Chao1, Shannon, observed species and Simpson demonstrate that the total number of bacteria and species richness of CCP were higher than those of CCN and OCP. It indicates that the closed circuit facilitated EAB to produce electrons, and the plants helped rhizosphere anode to enrich a large number of bacterial communities to increase electricity generation and gas production (e.g., CH_4_). Faith-pd and Pielou-e illustrate that the higher their values, the better the genetic diversity of the species and the homogeneity of the community. The above phenomenon is also reflected in Fig. 2 that the function of wetland plants contributed to promoting electricity production and increasing CH_4_ emission, mainly owing to the enhancement of the bacterial community. Of course, plant rhizosphere helps microorganisms (e.g., EAB) to adhere and obtain nutrients (e.g., root exudates), thus accelerating extracellular electron transfer (Zhang et al. 2016).

**Table 2:** α-Diversity indices of bacterial communities in the anode biofilms of different CW-MFCs

Sample	Chao1	Faith**-**pd	Observed**-**species	Pielou**-**e	Shannon	Simpson
CCP	3397.8	197.6	2849.2	0.79	9.05	0.99
OCP	1621.4	89.9	1325.0	0.51	5.23	0.82
CCN	2502.4	142.6	2004.6	0.63	6.87	0.91

**Fig. 6:**
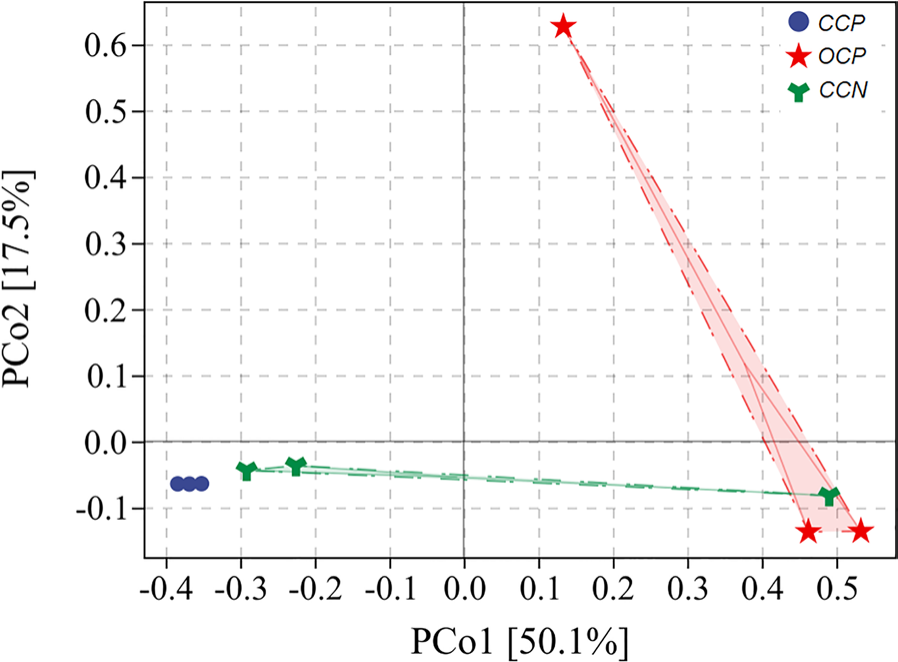
Distance matrix and PCoA analysis under different conditions in CW–MFCs

In the light of β Diversity analysis, principal co-ordinates analysis (PCoA) was carried out according to distance matrix, and the differences between flora were further expressed by dimensionality reduction, as shown in Fig. 6. Bray Curtis distance algorithm was used to represent the flora difference of EAB and methanogens in the CW–MFCs. It can be observed that there were similar EAB and methanogens between plant CW–MFC and non-plant CW–MFC under closed-circuit conditions. Because the distance between the coordinate axes was close, so the difference of flora was small. By comparing the open and closed-circuit CW–MFCs, the straight-line distance between samples was far from each other, so there were great differences in flora.

**Fig. 7:**
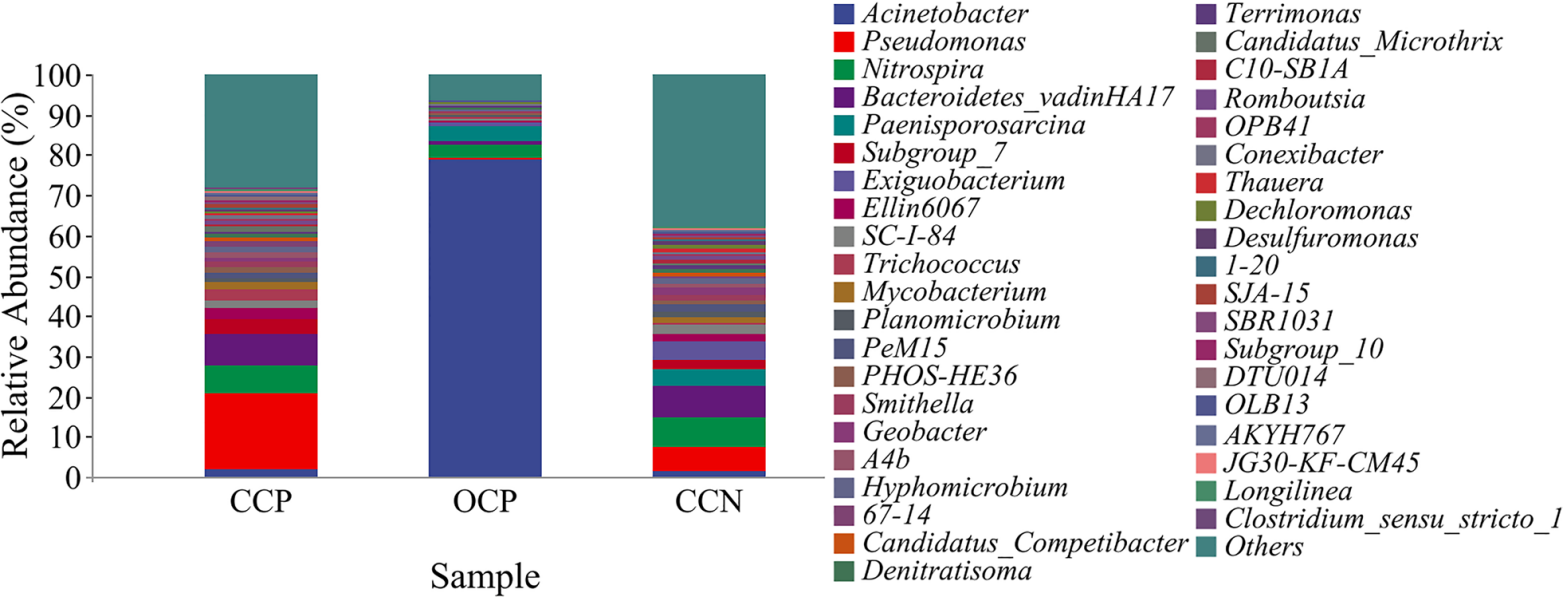
Relative abundance of major genera in CCP, OCP and CCN under 16S primers. Genera with relative abundances below the top forty are defined as “other”

As shown in Fig. 7, the effects of open/closed circuit and the presence/absence of plants on the anodic bacterial community were further investigated. In the open-circuit plant CW–MFC, the dominant flora consisted of *Acinetobacter*, *Paenisporosarcina* and *Nitrospira*, accounting for 78.77%, 3.51% and 3.29%, respectively. Among them, *Nitrospira* is a kind of nitrifying bacteria responsible for oxidizing ammonia nitrogen (Dai et al. 2019). In closed-circuit operation mode, the bacterial species richness of the plant CW–MFC was higher than that of the non-plant CW–MFC. The dominant flora of the plant CW–MFC included *Acinetobacter* (1.85%), *Pseudomonas* (18.77%), *Bacteroides* (7.68%), *Trichococcus* (2.62%), *Mycobacterium* (1.77%), *Smithella* (1.43%) and *Geobacter* (0.91%). On the basis of the above bacteria in the non-plant CW–MFC, the relative abundances were relatively small, accounting for 1.25%, 6.16%, 7.79%, 1.23%, 1.38%, 1.23% and 1.73%, respectively, but *Exiguobacterium* was endemic to the closed-circuit non-plant CW–MFC (4.47%). Among the above bacteria, the possible EAB included *Pseudomonas* (Pham et al. 2008), *Bacteroides* (Schamphelaire et al. 2008), *Mycobacterium* and *Geobacter* (Holmes et al. 2004). In addition, *Trichococcus* has good degradation of complex compounds and polysaccharides (Mielcarek et al. 2016), while *Smithella* mainly produces CH_4_ in the acid production stage (Puengrang et al. 2020). According to previous research, plants can affect the phylogeny of anodic EAB (Lu et al. 2015). For closed-circuit operation mode, the richness of microbial flora in the plant CW–MFC was higher than that in the non-plant CW–MFC, which might be because the addition of plants increased the diversity of flora. From the perspective of bacterial percentage, the relative abundances of EAB in the plant CW–MFC (e.g., *Pseudomonas* and *Mycobacterium* were 18.77% and 1.77%, respectively) were slightly higher than those in the non-plant CW–MFC (only 6.16% and 1.38%, respectively), which also greatly indicates that the plant rhizosphere improved nutrients for microorganisms, thus stimulating reproduction rate of EAB and making the power production of the plant group much higher than that of the non-plant group (Fig. 2B). Through comparative analysis between plant CW–MFCs under the conditions of open and closed circuit, it can be found that *Acinetobacter* was suitable to survive in the case of open circuit. The richness of microbial flora in closed circuit CW–MFC was higher than that in open circuit CW–MFC (Table 2), which further proves that the diversity of EAB in closed circuit plant CW–MFC was richer. As a result, the EAB and microbial electrogenesis processes greatly contributed to CH_4_ mitigation in the CW–MFC by functioning as a role of converting biogenic carbon into CO_2_ instead of CH_4_ under anaerobic conditions.

**Fig. 8:**
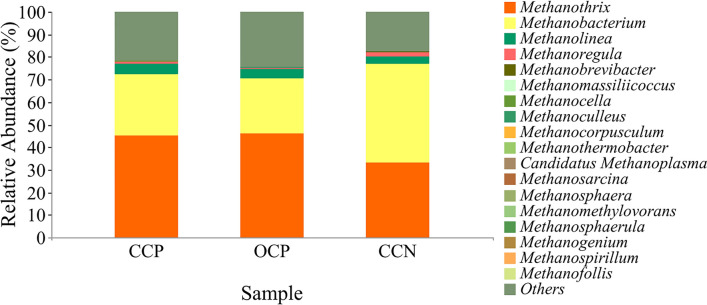
Relative abundance of major genera in CCP, OCP and CCN under methanogenic primers. Genera with relative abundances below the top twenty are defined as “other”

Methanogenic bacteria are the main genus of CH_4_ producing bacteria, and Fig. 8 shows that the main methanogens were *Methanothrix*, *Methanobacterium* and *Methanolinea*. Comparative analysis shows that the relative abundances of *Methanothrix*, *Methanobacterium* and *Methanolinea* in the open-circuit plant CW–MFC were approximately 1.00%, 2.79% and 0.36% higher than those in the closed-circuit plant CW–MFC, respectively. Therefore, the open circuit mode was favorable for methanogens to produce CH_4_. By comparing the presence and absence of plants in the closed-circuit CW–MFCs, it can be known that the relative abundance of *Methanothrix* in the plant group was approximately 4.74% higher than that in the non-plant group, which further proves that the CH_4_ emission flux of the closed-circuit non-plant CW–MFC was the lowest (Fig. 2A). However, only *Methanobacterium* was higher in the closed-circuit non-plant CW–MFC, with a value of approximately 2.44%, which might be because the *Methanobacterium* would favor easily decomposed carbon source (e.g., CO_2_ and formate) as nutrient, while cellulose is a macromolecular organic matter and difficult to be decomposed. The coexistence of *Methanothrix* and *MethanoRegula* can be concluded from the analysis, which indicates that CH_4_ production greatly originated from wetlands, and acetate and CO_2_/H_2_ were two commonly used substrates (Galand et al. 2005).

### Mechanism of CH_4_ emission

**Fig. 9:**
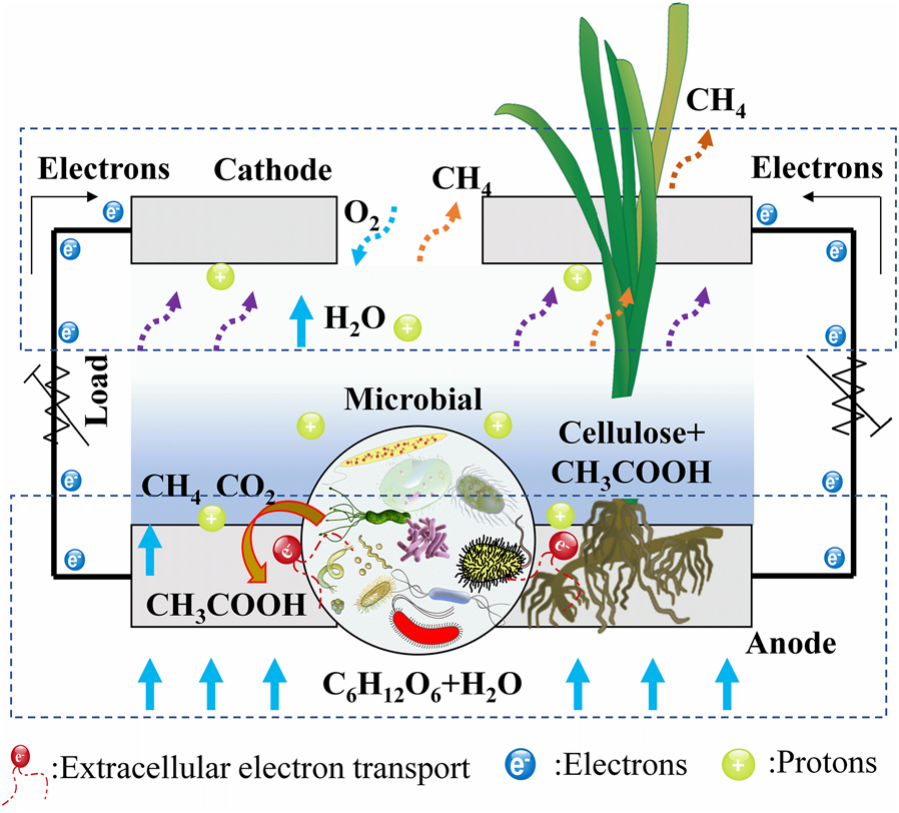
Mechanism diagram of carbon conversion and CH_4_ emission in the CW–MFC

To illustrate the principle of controlling CH_4_ emission in microbial electrogenesis process (Fig. 9), the competition mechanism between EAB and methanogens was explored from the above study (Figs. 7 and 8), as well as the analysis of bioelectricity generation and CH_4_ emission (Figs. 2 and 3). In general, CH_4_ is not produced under all environmental conditions, but rather anaerobic state of the wetland due to limited oxygen supply, which creates the prerequisites for wetland CH_4_ production (de la Varga et al. 2015). Of course, CH_4_ production is often divided into three distinct stages: (1) hydrolysis phase, the anaerobic fermentation and decomposition of complex organic substrates by fermenting bacteria into ethanol and fatty acids, etc.; (2) acid production phase, the production of acetic acid, hydrogen and CO_2_ by syntrophic bacteria or acetic acid production by specialized acetic acid-producing bacteria; (3) methanogenic phase, CH_4_ production from acetic acid or CO_2_/H_2_ in the presence of methanogenic bacteria (Chen et al. 2021b; Wang and Ren 2013). The specific equations are shown in Eqs. (8–12). The contributions of the two CH_4_ production pathways vary due to differences in microbial families, organic matter species and content, etc. in different wetlands. A study of peat bogs using isotope tracing found that 70% of the CH_4_ was produced by fermentation of acetic acid, while only 30% was formed by reduction of CO_2_ (Chen et al. 2021a):

8 $${\text{C}}_{{6}} {\text{H}}_{{{12}}} {\text{O}}_{{6}} + {\text{2H}}_{{2}} {\text{O}} \to {\text{2CO}}_{{2}} + {\text{2CH}}_{{3}} {\text{COOH}} + {\text{4H}}_{{2}}$$

9 $${\text{CH}}_{{3}} {\text{COOH}} \to {\text{CO}}_{{2}} + {\text{CH}}_{{4}}$$

10 $${\text{4HCOOH}} \to {\text{CH}}_{{4}} + {\text{3CO}}_{{2}} + {\text{2H}}_{{2}} {\text{O}}$$

11 $${\text{4CH}}_{{3}} {\text{OH}} \to {\text{3CH}}_{{4}} + {\text{CO}}_{{2}} + {\text{2H}}_{{2}} {\text{O}}$$

12 $${\text{CO}}_{{2}} + {\text{4H}}_{{2}} \to {\text{CH}}_{{4}} + {\text{2H}}_{{2}} {\text{O}}{.}$$

Microbial competition is a vital factor affecting CH_4_ emission from the CW–MFC. In the wastewater treatment by the CW–MFC, EAB compete with methanogens for carbon source and convert labile carbon directly into CO_2_ rather than CH_4_, which would greatly reduce the greenhouse effect contribution of wetlands to the global atmosphere, as CO_2_ has a much lower global warming potential than CH_4_ (Liu et al. 2017). The results of this study also verified that closed-circuit conditions (i.e., microbial electrogenesis) resulted in lower richness of methanogens (Fig. 8) and less emission flux of CH_4_ (Fig. 2A).

Plant physiological activity is another important factor influencing CW–MFC CH_4_ emission. Wetland plants can affect the bacterial community responsible for CH_4_ production in two opposite means: (1) producing and secreting organic carbon, and (2) transporting oxygen to rhizosphere (Silvey et al. 2019). Root exudates or plant biomass can promote the growth of methanogens and methanogenesis, thus increasing CH_4_ flux. Oxygen delivered by ventilatory tissue of plant roots can stimulate the growth of aerobic methane-oxidizing bacteria, thereby reducing CH_4_ flux. In typical constructed wetlands, oxygen released by plant roots can supply approximately 0.43–1.12% of biochemical oxygen demand (Zhang et al. 2014). In addition, oxygen released from plant roots varies widely and mainly depends on plant species, temperature, oxygen concentration and light intensity (Feng et al. 2022). Wetland plant can also affect CW–MFC electricity generation by influencing electrode-associated bacteria or electrochemical reactions. When cathode is located in plant rhizosphere, oxygen released by plant roots can be used as electron acceptor, which is beneficial to cathodic reaction and electricity generation (Liu et al. 2014). In this study, anode was located in plant rhizosphere and root deposits were used as the fuel, which substantially enhanced EAB richness (Fig. 7) and bioelectricity production (Fig. 2B), while cathode was located in overlying oxygen-rich water to facilitate oxygen diffusion. Plant activities, including transport of oxygen and release of root exudates, increase wetland environmental diversity and thus change rhizosphere microbial community, subsequently affecting the chemical and biochemical processes of CH_4_ emission (Feng et al. 2022). Nevertheless, different wetland plant species often have different effects on CH_4_ emission (Silvey et al. 2019), and the results of this study shows that wetland plants contributed to an increase in CH_4_ emission from the CW–MFC. Therefore, in the design and operation of the CW–MFC, appropriate strategies of plant selection (e.g., plant species that inhibit methanogenesis or with poor CH_4_ transport) and operational management (e.g., plant harvesting) should be adopted to minimize CH_4_ emissions. Furthermore, the potential of field-scale CW–MFC on CH_4_ emission should be explored in the future.

## Conclusions

Nowadays, GHG emissions continue to be severe and climate deterioration is still accelerating, reflecting strongly on environmental factors such as the atmosphere, water bodies, and soils. CW–MFCs provide direction and technical support for the development of carbon sink technologies, making control of GHGs such as CH_4_ a reality. The experimental results showed that wetland plant contributed to an increase in CH_4_ emission flux. By comparing different CW–MFCs, CH_4_ emissions can be effectively controlled under the conditions of low organic substrate concentration in influent and closed-circuit operation mode. High-throughput sequencing showed that anodic microorganisms differed significantly under different conditions. By changing environmental factors, anodic electrogenic bacteria can gain a superiority in nutrient competition, thereby reducing CH_4_ emissions from the CW–MFC. The CW–MFC technology has great potential in controlling CH_4_ emissions from wetlands, and moreover, the relationship between CH_4_ and CO_2_ emissions needs be further addressed.
